# Neuroprotective Effects of Phenothiazines on Acute Ischemic Stroke

**DOI:** 10.1002/cns.70960

**Published:** 2026-06-09

**Authors:** Qi Wang, Weili Li, Di Wu, Shuaili Xu, Xunming Ji

**Affiliations:** ^1^ Department of Neurosurgery, Xuanwu Hospital Capital Medical University Beijing China; ^2^ Department of Neurology and China‐America Institute of Neuroscience, Xuanwu Hospital Capital Medical University Beijing China; ^3^ Department of Neurology The First Affiliated Hospital of Shandong First Medical University & Shandong Provincial Qian Fo Shan Hospital Jinan China; ^4^ Beijing Institute of Brain Disorders, Laboratory of Brain Disorders, Ministry of Science and Technology, Collaborative Innovation Center for Brain Disorders Capital Medical University Beijing China

**Keywords:** chlorpromazine and promethazine, ischemic penumbra, neuroprotective effects, phenothiazines

## Abstract

**Background:**

Standard therapeutic strategies for acute ischemic stroke (AIS) include endovascular mechanical thrombectomy, intravenous thrombolysis, bridging therapy, and conventional conservative treatment. Despite the implementation of standardized treatments, nearly half of patients still experience significant motor or speech dysfunction, severely impacting their quality of life. Consequently, there is a pressing need for effective neuroprotective adjunctive therapies to further improve functional outcomes and prognosis. Phenothiazines, primarily chlorpromazine and promethazine (C + P), have been proposed as potential neuroprotective agents. Preclinical evidence suggests that phenothiazines may induce mild hypothermic responses, exert anti‐inflammatory effects, and promote a hypometabolic state, thereby potentially contributing to improved neurological outcomes.

**Methods:**

We reviewed and synthesized the mechanisms underlying the neuroprotective effects of phenothiazines, with a particular focus on C + P.

**Results:**

This review highlights the neuroprotective mechanisms of phenothiazines related to mild hypothermia, anti‐inflammation, and metabolic reduction, along with relevant preclinical and clinical evidence. We also compare the distinct mechanisms of action between C + P and conventional anesthetic sedatives and draw partial comparisons with natural hibernation. Our findings reveal that C + P possesses unique features and advantages in neuroprotection, particularly regarding metabolic suppression and hypothermia.

**Conclusions:**

This review explores the adjunctive role of phenothiazines following AIS and elucidates their modulatory mechanisms, which may exert neuroprotective effects through multiple pathways and potentially improve AIS outcomes. This approach may facilitate the identification of novel specific targets for conventional drugs, thereby offering a new perspective on addressing the challenges associated with AIS.

## Graphical abstract

For acute ischemic stroke due to large vessel occlusion, adjunctive neuroprotective therapy with mechanical thrombectomy is important; however, current treatment options are relatively limited. Phenothiazines such as chlorpromazine and promethazine (C + P) have shown potential neuroprotective effects in preclinical studies. This review describes several reported mechanisms that may underlie the neuroprotective actions of C + P, thus offering a new perspective on pharmacological neuroprotection as an adjunctive therapy.

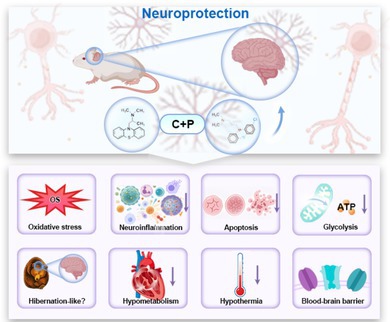

## 1 Introduction

Acute ischemic stroke (AIS) represents a major global health burden and is among the leading causes of morbidity and mortality, often resulting in a substantial decline in quality of life [1]. Following the onset of AIS, a cascade of pathological events occurs, including reduced cerebral perfusion, generation of oxygen‐free radicals, significant alterations in neurotransmitter and ion homeostasis, and accumulation of toxic metabolites in affected brain regions. These processes contribute to extensive necrosis within the ischemic core, ultimately leading to impaired neurological function [2]. Current management of AIS involves a multimodal approach combining pharmacotherapy, endovascular interventions, and rehabilitation, as outlined in established guidelines and consensus statements. Antithrombotic agents have long served as a cornerstone of ischemic stroke treatment and are frequently administered alongside intravenous thrombolysis with recombinant tissue plasminogen activator (rt‐PA) and mechanical thrombectomy to facilitate reperfusion [3]. With ongoing advances in therapeutic techniques and the extension of treatment time windows, recanalization rates for acute large vessel occlusions have improved to approximately 70%. Despite timely and comprehensive treatment, however, 40%–50% of patients still experience severe functional disabilities, with some facing life‐threatening complications [4]. Thus, effective adjunctive therapies to mitigate neurological injury and improve clinical outcomes in AIS patients are urgently needed [5].

Neuroprotective therapy in the acute phase and functional recovery following neurological injury represent critical challenges in the management of AIS, necessitating further investigation in therapeutic research [6]. The ischemic penumbra—a region of functionally impaired but potentially salvageable tissue surrounding the ischemic core—serves as a key target for acute‐phase neuroprotection. Salvaging the penumbra and reducing the final infarct volume are pivotal for improving clinical outcomes in AIS patients. Effective intervention can attenuate the expansion of the ischemic penumbra or limit infarct growth, thereby enhancing functional recovery after neurological injury. Recent evidence indicates that in patients with large artery occlusion, the ischemic penumbra shrinks by approximately 3 mL per hour after stroke onset [7], highlighting the time‐sensitive nature of penumbral loss and the progressive reduction of salvageable tissue. Therefore, in addition to endovascular recanalization, adjunctive pharmacologic therapies that can halt penumbral progression, preserve viable brain tissue, and ultimately minimize neurological impairment and improve treatment efficacy in AIS patients are needed.

Phenothiazines, notably C + P, have shown potential neuroprotective properties in the context of post‐reperfusion recovery after AIS [8, 9]. Early clinical studies reported significant improvements in both short‐ and long‐term neurofunctional scores with their use. However, safety concerns and a range of adverse effects—such as hypotension, pulmonary infections, intracranial hemorrhage, and exacerbation of pre‐existing conditions—have limited their clinical adoption. These issues may be partly attributable to historical limitations in medical technology, including suboptimal drug administration techniques, uncontrolled dosing ratios, infusion rates, and inadequate vital sign monitoring. With recent advances in clinical monitoring and treatment protocols, as well as renewed interest in phenothiazines, this therapeutic strategy has attracted increasing attention in the field of neuroprotection [8, 10]. In recent years, numerous rodent studies have reported that C + P administration is associated with reduced infarct volume and improves neurobehavioral outcomes in models of cerebral ischemia. Nevertheless, clinical evidence remains scarce, and the therapeutic efficacy of C + P in human AIS patients has yet to be conclusively established. Thus, the role of C + P in clinical practice warrants further rigorous investigation.

## 2 Neuroprotective Mechanisms of Phenothiazines

**FIGURE 1:**
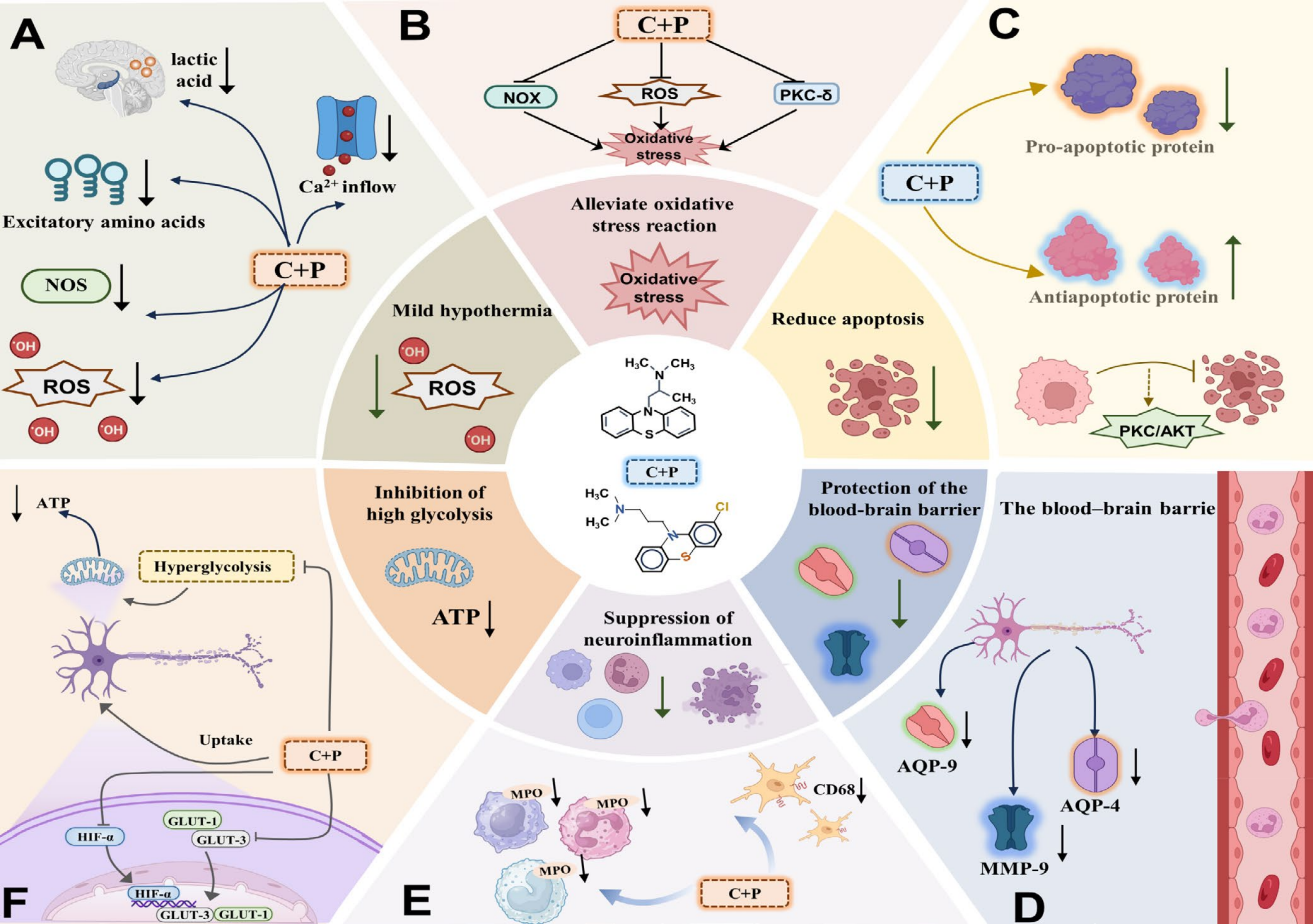
The neuroprotective mechanism of C + P. (A) Inducing mild hypothermia is the primary mechanism through which C + P exerts its neuroprotective effect. (B) C + P may counteract oxidative stress processes through the PKC‐delta‐NOx‐Rox pathway. (C) C + P can reduce apoptotic injury in ischemic stroke by acting on related proteins through the PKC/AKT pathway. (D) C + P protects the blood–brain barrier by acting on the targets of MMP‐9, AQP‐4, and AQP‐9. (E) C + P suppresses inflammation by reducing MPO and CD68 levels to inhibit immune cell infiltration and apoptosis. (F) C + P works through the GLUT‐3 and PFK‐1 pathways to reverse ATP consumption and improve energy consumption by altering glucose metabolism.

A comprehensive understanding of the mechanisms underlying the effects of phenothiazines, primarily C + P, is essential for leveraging their neuroprotective properties and exploring novel adjunct treatment strategies. Accumulating evidence from animal studies has supported the neuroprotective efficacy of C + P in AIS models, revealing several interconnected mechanisms (Figure 1). The neuroprotective effects of C + P in experimental stroke models have been attributed to a combination of pharmacological actions, hypothermic effects, and metabolic suppression. However, these mechanisms are often intertwined in experimental settings, making it challenging to disentangle temperature‐dependent from temperature‐independent components. We review the current evidence organized according to three distinct but potentially overlapping categories: Direct pharmacological effects, hypothermia‐mediated protection, and metabolic suppression. We then discuss how these mechanisms relate—conceptually, rather than equivalently—to the naturally occurring hibernation state.

### 2.1 Pharmacological Effects Independent of Temperature Reduction

One way to assess whether C + P has intrinsic neuroprotective effects independent of hypothermia is to examine experiments in which core temperature is artificially maintained at normothermic levels. Under these conditions, several studies have reported reductions in infarct volume and improvements in neurological outcomes that cannot be attributed simply to cooling [11, 12].

#### 2.1.1 Inflammatory Responses

The inflammatory cascade represents a key pathophysiological response activated after AIS; although inflammation serves as a physiological defense mechanism, it can significantly exacerbate brain injury, particularly during the acute and subacute phases of cerebral ischemia. Phenothiazines have been demonstrated to attenuate this inflammatory response, thereby conferring protection to compromised brain tissue [9, 13]. In temperature‐controlled Middle cerebral artery occlusion (MCAO) models, C + P administration was associated with reduced expression of myeloperoxidase (MPO) and the microglial marker CD68 at 6 h and 24 h after reperfusion [14]. Both markers are elevated during post‐ischemic inflammation; their attenuation under normothermic conditions suggests that C + P may inhibit immune cell infiltration and apoptotic cell death through pathways not requiring temperature reduction. Additional evidence points to suppression of JAK2/STAT3 and p38 phosphorylation, along with reduced HIF‐1α protein expression, under temperature‐controlled preparations [15].

#### 2.1.2 NLRP3 Inflammasome and HIF‐1α

The NLRP3 inflammasome, a key mediator of neuroinflammation, has been examined in relation to C + P treatment under different thermal conditions. When core temperature was maintained at 37°C, C + P treatment reduced HIF‐1α expression, with subsequent attenuation of mitochondrial dysfunction and NLRP3 inflammasome activation. In vitro experiments further indicated that these normothermic effects are mediated primarily through HIF‐1α rather than temperature‐sensitive pathways [12]. NLRP3 inflammasome activation is known to depend on RIPK1 kinase activity, a process critically involved in the generation of key inflammatory cytokines. In parallel, HIF‐1α contributes to the regulation of essential biological processes, including inflammation and programmed cell death. Geng et al. demonstrated that C + P significantly reduced the protein expression of the NLRP3 inflammasome complex, and further emphasized that under hypothermic conditions, C + P primarily exerts its effects via the RIPK1/RIPK3‐DRP1 signaling pathway [12].

#### 2.1.3 Oxidative Stress

Phenothiazines have long been recognized for their free radical scavenging properties [16]. In the context of stroke, C + P appears to modulate protein kinase C‐δ (PKC‐δ) and nicotinamide adenine dinucleotide phosphate oxidase (NOX), both of which contribute to oxidative injury [5]. Treatment with C + P has been associated with reduced post‐reperfusion reactive oxygen species levels, decreased NOX activity, downregulation of NOX subunits, and interference with the interaction between PKC‐δ and the cytosolic subunit p47phox^11^. These effects were observed even under conditions in which hypothermia was not allowed to contribute. After AIS onset, the accumulation of harmful substances, including free radicals, contributes to mitochondrial dysfunction and neuronal peroxidation damage. Oxidative stress represents one of the fundamental pathophysiological processes in AIS, and by modulating these key signaling proteins, C + P helps prevent the expansion of the ischemic penumbra, reduce infarct volume, and improve neurofunctional scores in MCAO mouse models [11].

#### 2.1.4 Apoptosis Regulation

Ischemia–reperfusion injury activates apoptotic cascades, and the PKC/AKT signaling pathway has been implicated in counteracting this process [17, 18]. In rodent stroke models, C + P treatment was associated with increased expression of antiapoptotic proteins and decreased levels of pro‐apoptotic proteins at 6 h and 24 h after reperfusion, effects that were linked to modulation of the PKC/AKT pathway [19]. The PKC/AKT signaling pathway serves as a critical regulator of apoptosis, with activation promoting cellular differentiation and proliferation, inactivating multiple proapoptotic factors, and exerting antiapoptotic effects. Research on ischemia–reperfusion injury indicates that PKC/AKT‐related proteins are extensively involved in the regulation of cell death and survival, and this pathway contributes to a reduction in oxidative stress while supporting the recovery of neurological function [17, 18, 20].

#### 2.1.5 Blood–Brain Barrier Integrity

The integrity of the blood–brain barrier is critically compromised following AIS, largely due to the mechanisms of edema formation. This breakdown disrupts the homeostasis between brain tissue and cerebral blood flow, contributing to a range of neurological deficits. MMPs and AQPs are key contributors to blood–brain barrier disruption in stroke and have emerged as potential pharmacological targets for neuroprotection [21].

Matrix metalloproteinases (MMPs) and aquaporins (AQPs) contribute to blood–brain barrier disruption after stroke [21]. In MCAO mice receiving C + P (8 mg/kg), expression of MMP‐9, AQP‐4, and AQP‐9 was reduced, and infarct volume at 48 h post‐reperfusion was decreased by 35.7% even when normothermia was maintained. Notably, the reduction was more pronounced (29.8%) when temperature was not controlled, suggesting that hypothermia adds an additional protective effect. The persistence of blood–brain barrier protection beyond the 6‐h duration of drug‐induced hypothermia further points to a hypothermia‐independent component [11].

### 2.2 Protective Mechanisms Linked to Hypothermia

The hypothermic effect of C + P has been characterized in some detail. In rat MCAO models, intraperitoneal injection at 8 mg/kg produces a temperature decrease beginning within 5 min, reaching 34.1**°C** after approximately 2 h, and persisting for about 6 h before gradually returning to baseline [22]. C + P exhibits sedative properties, reduces systemic blood flow, decreases oxygen consumption, dilates blood vessels, and induces mild hypothermia [22, 23]. Previous research has shown that for every 1**°C** decrease in body temperature, the metabolic rate of the brain decreases by approximately 6%–7% [24]. A moderate reduction in cerebral metabolic rate contributes to neurologic recovery. The therapeutic effect of mild hypothermia has been extensively studied and widely applied in cases of cardiac arrest and cerebral ischemic diseases [25].

Mild hypothermia itself is known to reduce cerebral metabolic rate by approximately 5%–7% per 1**°C** decrease [24, 26]. It also helps preserve blood–brain barrier integrity, alleviates brain edema, and lowers intracranial pressure [25, 26]. Additionally, mild hypothermia inhibits the release of excitatory amino acids, decreases nitric oxide synthase activity, limits Ca^2+^ influx, and suppresses the generation of oxygen free radicals while promoting their clearance. At the molecular level, it downregulates the expression of the immediate early gene c‐fos, reduces the release of inflammatory factors, and suppresses neuronal apoptosis [25, 26]. In rodent models, every 1**°C** reduction in core temperature leads to a 5% decrease in brain metabolism, and when the temperature decreases to 33**°C**, it can reduce the volume of damaged brain tissue by 30% [26].

More recent work has identified pathway‐specific temperature dependence. At 33°C, C + P treatment was associated with suppression of RIPK1, RIPK3, DRP‐1, NLRP3 inflammasome components, and cytochrome c‐mediated apoptosis, suggesting that under hypothermic conditions, the drug acts primarily through the RIPK1/RIPK3–DRP1 axis. By contrast, at maintained normothermia (37°C), HIF‐1α emerged as the dominant mediator. These findings indicate that the same drug may engage distinct molecular pathways depending on the thermal environment [12].

The timing of hypothermia induction may influence outcomes. Preclinical studies have suggested that initiating cooling early during the ischemic phase, ideally before or concurrent with reperfusion, may maximize salvage of the ischemic penumbra [27, 28, 29]. In animal experiments, more than half of the studies initiated hypothermia within one hour of stroke onset, and an additional 20% began cooling before ischemia induction [30]. The rapid onset of C + P‐induced cooling—within 5–10 min—contrasts with the 30 min typically required for physical cooling alone [31]. Early hypothermia induced by low body temperature combined with dihydrocapsaicin (DHC)/phenothiazine can inhibit harmful gluconeogenesis, improve glucose metabolism, and reduce lactate and oxidative stress responses after acute stroke. Unlike inherent temperature regulation mechanisms, C + P allows for a more stable and rapid reduction in body temperature, and higher doses of chlorpromazine (10 mg/kg) can be used alone for low‐temperature induction in rat models [22].

When C + P was combined with physical cooling in MCAO models, infarct reduction was greater than with either intervention alone (*p* < 0.001), whereas neither intervention alone produced a statistically significant effect in some study designs (*p* = 0.85 and *p* = 0.61, respectively) [32, 33]. These observations raise the possibility that C + P may serve as an adjunct to physical cooling strategies, though the magnitude of synergy likely depends on experimental conditions. In MCAO rodent models, significant reductions in ischemia‐mediated pathological changes in tissue were observed after hypothermia induction, with the greatest reduction in infarction volume reaching 90% [34].

### 2.3 Metabolic Suppression as a Contributing Factor

AIS disrupts cerebral glucose metabolism, with accelerated glycolytic flux and upregulation of the glucose transporters GLUT‐1 and GLUT‐3 in affected brain regions [35]. Enhanced lactate dehydrogenase activity then converts pyruvate to lactate, contributing to tissue acidosis and cellular injury [36]. Early investigations indicated that C + P can attenuate cerebral glucose uptake and utilization, thereby suppressing glycolysis, ameliorating the severity of acidosis, and mitigating brain damage, although the precise mechanism underlying these effects remains elusive [37].

C + P treatment has been associated with downregulation of GLUT‐1, GLUT‐3, and HIF‐1α expression [38]. When temperature was not controlled, improvements in glucose metabolism, NOX activity, ATP levels (at 6 h post‐reperfusion), and GLUT‐1 expression (at 24 h) were more pronounced than under temperature‐controlled conditions. Nevertheless, some metabolic effects persisted even when normothermia was maintained, suggesting that modulation of cerebral glucose metabolism may occur partially independent of hypothermia [14].

Within neurons, glucose transport is facilitated primarily by GLUT‐3, and phosphofructokinase‐1 (PFK‐1) serves as a rate‐limiting enzyme in glycolysis [39]. C + P appears to interact with both pathways, with associated reversal of ATP depletion and improvement in energy utilization efficiency [40]. This metabolic reprogramming contributes to attenuated neuronal damage and represents a key hypothermia‐independent mechanism underlying C + P neuroprotection.

As outlined previously, the cerebral response to AIS involves compensatory upregulation of glycolysis in an effort to sustain energy homeostasis; however, this adaptive mechanism proves insufficient to meet cellular energy demands and paradoxically contributes to detrimental outcomes, including lactic acidosis and excessive reactive oxygen species production, which collectively exacerbate ischemic brain injury [40].

Under C + P‐induced hypometabolism, succinyl‐CoA ligase activity may lead to succinic acid accumulation during cerebral hypoperfusion, with possible implications for limiting reactive oxygen species generation. Concurrent suppression of mitochondrial oxidative metabolism may further restrict ROS production [41]. Phenothiazines have also been reported to attenuate free radical‐induced lipid peroxidation and stabilize mitochondrial membranes, potentially inhibiting cytochrome c release and subsequent caspase‐dependent apoptosis [42]. The observation that phenothiazines inhibit the cellular uptake of non‐metabolizable glucose analogs further suggests that their primary action involves glucose transport machinery rather than intracellular metabolic pathways [43].

This metabolic reprogramming contributes to attenuated neuronal damage and represents one of the key hypothermia‐independent mechanisms underlying the neuroprotection conferred by C + P. The degree of metabolic suppression achieved can be substantial, with estimates indicating that ATP demand may decrease by as much as 70%–80% under such conditions. Cerebral metabolism declines by approximately 5% for each 1°C reduction in tissue temperature, a relationship that has been documented in hibernating animal models [38, 44].

Additionally, phenothiazines are associated with significant reductions in cerebral blood flow and oxygen consumption [45]. Although their precise molecular targets remain under investigation, proposed mechanisms include minor mediation via D2 dopamine receptor antagonism and a potentially more prominent role involving the modulation of voltage‐gated Ca^2+^ channels [46].

### 2.4 Distinctions Between Drug‐Induced Protection and True Hibernation

Reduced body temperature and suppressed metabolic rate are two defining features of natural hibernation [10]. In some hibernating species, cerebral blood flow can decrease to less than 10% of active levels, while glucose utilization falls to below 2% [47]. Heart rate and blood pressure also decline substantially [47, 48], and the hibernation phenotype is associated with coordinated changes in hundreds of genes and metabolites [47].

The neuroprotective capacity of natural hibernation has prompted interest in whether a “hibernation‐like state” can be induced pharmacologically as a therapeutic strategy for acute brain injury [49, 50, 51]. C + P has been discussed in this context because it produces two features that are also characteristic of hibernation: Reduced body temperature and decreased metabolic demand. This state is distinct from therapeutic hypothermia; it represents a broader, coordinated physiological paradigm in which hypothermia is one component, but sustained hypometabolism and specific receptor signaling (e.g., central adenosine A1 receptors) are crucial for maintaining vital functions such as normal sinus rhythm during cooling [50].

Whether C + P induces a state that is truly analogous to hibernation remains an open question. Hibernation is an endogenously orchestrated, temporally regulated process involving systemic adaptations across multiple organ systems. The effects of C + P, by contrast, represent an externally imposed, pharmacologically driven condition that reproduces only selected features of the hibernation phenotype. Available evidence does not suggest that C + P activates the full molecular program of hibernation [52, 53].

Chlorpromazine itself exerts neuroprotection through multiple mechanisms–including vasodilation, temperature reduction, and dopamine receptor‐mediated inhibition of glucose metabolism—that together initiate a protective state distinct from true hibernation [52]. The independent neuroprotective role of the C + P combination is increasingly recognized as a distinct area of investigation rather than a recapitulation of hibernation biology [53].

Chlorpromazine and promethazine are individually FDA‐approved medications, and their combination has been widely used in some countries for cooling, sedation, and postoperative neuroprotection in patients with craniocerebral trauma. In rat MCAO models, administration of C + P (each at 10 mg/kg) has been associated with reduced infarct volume, decreased cellular apoptosis at 48 h, and improved neurological function scores [32, 54, 55]. The neuroprotective efficacy of this combination is well‐established, with promethazine itself being identified as a neuroprotective agent as early as 2004. Furthermore, combined therapy with chlorpromazine and promethazine has been demonstrated to induce a hypometabolic state in rats, significantly reducing cerebral infarction volume and improving neurological function scores in MCAO models [30].

Nonetheless, the term “hibernation‐like” may warrant restrained use. Distinguishing between a drug‐induced protective state and true hibernation—conceptually and terminologically—may help avoid overinterpretation of the mechanisms at play. As molecular dissection of C + P action continues, a clearer framework for categorizing temperature‐dependent and temperature‐independent pathways will likely inform the design of future translational studies [12].

### 2.5 Distinctions Between C + P and Conventional Anesthetics/Analgesics

A point that deserves particular emphasis is that most anesthetics/sedatives (e.g., propofol, benzodiazepines, dexmedetomidine, barbiturates) can also induce hypothermia or metabolic suppression, but this is not exactly the same as the mechanism of action of C + P [8, 56].

First of all, the hypothermia and hypometabolism caused by most anesthetics/sedatives are thought to be passive consequences of central nervous system depression. After extensive suppression of brain activity, the thermoregulatory set point is not actively reset. The body continues to resist cooling through mechanisms such as shivering and vasoconstriction [56]. As a result, additional interventions—such as neuromuscular blockers or physical cooling—are often required to achieve hypothermia in clinical practice. This state appears fragmented and uncoordinated. By contrast, the state induced by C + P is different. C + P act on dopamine D2 receptors, histamine H1 receptors, and α‐adrenergic receptors, actively resetting the hypothalamic thermoregulatory set point while simultaneously suppressing nonshivering thermogenesis and vasoconstrictive responses [5, 45]. This results in rapid cooling within 5–10 min without shivering [40]. In physiological terms, this pattern of rapid, smooth, nonopposing cooling may more closely resemble the active entry into hypothermia seen in hibernating animals, which also actively lower their set point and suppress thermogenesis during torpor entry. Thus, the state induced by C + P is not simply “forced hypothermia” but rather an active, coordinated, and procedural hypothermic state [30].

Second, the mechanisms of conventional anesthetics/sedatives are highly concentrated: Propofol and barbiturates enhance GABA‐A receptor function; ketamine antagonizes NMDA receptors; dexmedetomidine activates α_2_ receptors [56]. The result is widespread neuronal suppression, and the hypothermia and hypometabolism are the secondary mathematical outcome of this inhibition, rather than programmed adaptive responses. C + P appears to differ in fundamental respects. It acts simultaneously on neural, metabolic, vascular, immune, and mitochondrial systems, and many of its effects have been observed under normothermic conditions in experimental models, suggesting they may be independent of hypothermia [12, 23]. These effects include: Suppression of the NLRP3 inflammasome and HIF‐1α (anti‐inflammatory) [12]; inhibition of the PKC‐δ/NOX pathway (antioxidant) [23]; Downregulation of MMP‐9, AQP‐4, and AQP‐9 (blood–brain barrier preservation) [22]; inhibition of GLUT‐1/GLUT‐3 and PFK‐1 (glucose metabolism reprogramming) [46]. Furthermore, promethazine can also directly stabilize the mitochondrial membrane and inhibit the release of cytochrome c (acting as an anti‐apoptotic agent) [55]. This multitarget, hypothermia‐independent cytoprotective network is rarely reported with conventional sedatives [5, 23].

In addition to hypothermia and hypometabolism, C + P also recapitulates several other specific features: (i) rapid cooling without shivering (suppression of nonshivering thermogenesis) [40]; (ii) maintenance of cardiovascular stability and normal rhythm at low temperatures [45]; (iii) metabolic reprogramming prioritizing alternative substrates [46]; and (iv) cellular resistance to ischemia reperfusion injury [55]. Standard anesthetics generally lack these features. For example, C + P induces hypothermia within 5–10 min through central dopaminergic and histaminergic pathways, whereas physical cooling or anesthetic‐induced hypothermia typically requires more than 30 min and frequently triggers shivering [40]. C + P also reduces cerebral oxygen consumption and blood flow in a pattern distinct from that seen in barbiturate‐induced coma. Furthermore, in experimental models, the synergistic reduction in infarct volume observed when C + P is combined with physical cooling cannot be explained by sedation alone, suggesting the activation of endogenous protective pathways [32].

In contrast, most conventional sedatives lack the ability to simultaneously suppress inflammation, preserve blood–brain barrier integrity, and reprogram glucose metabolism under hypothermia‐ independent conditions [23]. They cannot achieve rapid cooling without shivering or cardiovascular instability, lack cytoprotective mechanisms independent of central nervous system depression, and fail to reproduce the unique metabolic reprogramming (e.g., the shift from glucose to fatty acid/ketone utilization) seen with C + P^55^. Therefore, the hypothermia and hypometabolism, or other phenotypic features induced by C + P, are not merely “hibernation‐like” at the phenomenological level. At the cellular and molecular levels, they may also activate protective responses similar to those observed in true hibernation—a capacity that conventional anesthetics alone do not appear to possess.

## 3 Preclinical Study on the Neuroprotective Effect of Phenothiazine

The neuroprotective effects of phenothiazines have been extensively confirmed in numerous animal studies. These assessments primarily rely on evaluating changes in the ischemic penumbra or infarct volume via Triphenyltetrazolium Chloride (TTC) staining of brain tissue sections, coupled with improvements in neurological function scores. Most of these studies use a unilateral MCAO model, with C + P administered via intraperitoneal or intravenous injection. In mice, a common protocol involves intraperitoneal injection of C + P at a weight‐based dose (typically 8 mg/kg). Subsequent evaluations consistently revealed a reduction in cerebral infarct volume in the treatment group. C + P has been reported to exert neuroprotective effects in murine models, including attenuation of neuroinflammation, reduction of cellular apoptosis, and improvement in neurological function scores. These positive preclinical findings provide encouraging support for further clinical research and potential applications Table 1.

**TABLE 1:** Summary of preclinical studies on neuroprotective effects of C + P.

Study	Occlusion	Subject model	Measure	Dose	Core body temp	Infarct volume (%)	Functional outcome
Liu et al. 2015	2 h MCAO	54 adult Male rats	C + P	1.0 mg/kg	35.3°C ± 0.23°C	30.0 ± 5.35 (Reduced)	Improved
Geng et al. 2017	2hMCAO/4hMCAO	272 adult male rats	C + P	8 mg/kg	34.0°C/35.7°C	30.08 ± 2.57/36.42 ± 2.84 (Reduced)	Improved
An et al.2017	2hMCAO	30 rats	C + P&MH	—	33.4°C ± 0.6°C	30.7% ± 3.4% (Reduced)	Improved
Li et al. 2018	2 h MCAO	120 adult male rats	C + P	8 mg/kg	33°C–34°C	29.8–35.7 (Reduced)	Improved
Tong et al. 2019	2 h MCAO	64 adult male rats	C + P/NOX inhibitor	2.6 mg/kg	—	33.7% ± 6.0%/28.5% ± 3.0% (Reduced)	—
Guan et al. 2019	2 h MCAO	32 adult male rats	C + P	8 mg/kg	33.97°C ± 0.07°C	33.69 ± 6.03 (Reduced)	Improved
Guo et al. 2021	2 h MCAO	72 adult male rats	C + P	8 mg/kg	—	Reduced	—
Guo et al. 2021	2 h MCAO	126adult male rats	C + P	8 mg/kg	34.1°C	—	—
Han et al. 2021	2 h MCAO	32 adult male rats	C + P & DHC	4 mg/kg	↓3.75°C	32.1 (Reduced)	Improved
Geng et al. 2021	2 h MCAO	168 adult male rats	C + P & PCK inhibitor	4 mg/kg	—	30%	Improved
Guo et al. 2022	2 h MCAO	142 adult male rats	C + P & NOX inhibitor&PCK‐δ inhibitor	8 mg/kg	34°C	Reduced	Improved
Geng et al. 2024	2 h MCAO	150 adult male rats	C + P & HIF‐1α inhibitor	8 mg/kg	33°C	Reduced	Improved
Lv et al. 2023	2 h MCAO	49 adult male rats	C + P	4 mg/kg	Reduced	—	—

Abbreviations: 2hMCAO, The stroke groups underwent MCAO for 2 h at which point they were reperfused; C + P, Chlorpromazine and promethazine; DHC, dihydrocapsaicin; MCAO, Middle cerebral artery occlusion; MH, mild hypothermia; PCK, Phosphoenolpyruvate carboxykinase.

In a mouse model of unilateral MCAO, early intervention with a fixed dose of C + P was associated with a reduction in infarct volume compared to the non‐intervention group, as evidenced by analysis of brain tissue sections. This protective effect was particularly pronounced at 24 h post‐reperfusion, showing a 32% decrease in infarct volume. Concurrently, a significant reduction in reactive oxygen species (ROS) generation was observed at 6 and 24 h [33, 47], suggesting that C + P treatment may confer protection to the ischemic penumbra following stroke. Furthermore, numerous studies assessing neurological function scores have confirmed that compared with control treatment, C + P intervention leads to significant improvement in murine models of AIS at 48 h post‐treatment (*p* < 0.05) [20]. Consistent with these findings, Tong and colleagues reported that at 48 h after reperfusion, C + P treatment significantly reduced the infarct area in ischemic rats to 23.1% ± 5.5%, compared with 39.1% ± 3.1% in the control group (*p* < 0.05), findings that collectively support a neuroprotective effect in this model [54].

As previously discussed, drug‐induced sedation or hypothermia is currently regarded as the primary mechanism underlying this neuroprotective effect. However, a substantial body of research suggests that C + P may also possess independent neuroprotective properties distinct from those of hypothermia induction. To elucidate the contribution of hypothermia, many studies have implemented experimental designs comparing a blank control group, a C + P group with the core temperature maintained at 37°C, and a drug‐treated group without temperature control. Results consistently demonstrate that the non‐temperature‐controlled, drug‐treated group exhibits significantly lower neurological deficit scores and smaller infarct areas at 6 and 24 h compared to other groups (*p* < 0.01) [47, 57]. Moreover, even under temperature‐controlled conditions (37°C), C + P treatment significantly reduced apoptotic cell death after 48 h of reperfusion compared to the sham surgery group, as measured by ELISA. It was also observed that C + P significantly decreased the levels of the inflammatory factors MPO and CD68 in neutrophils and macrophages at 6 and 24 h post‐reperfusion (*p* < 0.001). Additional findings indicate that C + P can ameliorate reperfusion injury and reduce cerebral metabolism through other pathways [57]. Collectively, this evidence suggests that the neuroprotection afforded by C + P is multifaceted and may, at least in part, occur independently of hypothermia.

Substantial evidence supports the existence of independent neuroprotective functions for the C + P combination. A critical question that arises is whether the protective effects of C + P and mild hypothermia therapy are additive when applied concurrently. Given the established capacity of C + P to induce hypothermia and accelerate the achievement of target cooling temperatures, it is theoretically positioned to act as a synergistic adjunct to enhance the efficacy of mild hypothermia treatment. This potential synergy could significantly influence future clinical strategies for neuroprotection. Empirical findings corroborate this notion. One study reported that combined therapy was associated with a reduced infarct area compared to conventional treatment (*p* < 0.001). Notably, this effect was not observed in groups receiving either mild hypothermia or C + P as a standalone intervention (*p* = 0.85 and *p* = 0.61, respectively) [32, 33]. These results strongly indicate that the neuroprotective effect is uniquely evident under the combined treatment. However, the comparative efficacy of the individual treatment modalities remains a subject of debate, underscoring a vital direction for future research [9].

In recent years, significant research efforts have been directed toward identifying effective neuroprotective agents that can serve as straightforward and efficient adjunctive therapies in clinical practice. This focus has led to a consistent increase in the number of preclinical studies. Among the candidates, C + P has attracted considerable attention. A growing number of its underlying mechanisms and molecular targets—associated with anti‐inflammatory, antioxidant, blood–brain barrier preservation, and metabolic modulation—are being progressively elucidated [58]. These pathways are expected to become a major focus of future research. It is important to note, however, that most current preclinical evidence is derived from animal models involving transient ischemia (e.g., 2 h) followed by reperfusion. Consequently, any discussion of the neuroprotective mechanisms and efficacy of C + P must carefully consider the critical context of reperfusion.

## 4 Current Status of Clinical Application

In clinical practice, phenothiazines are most extensively utilized for sedation and analgesia. Their application in central nervous system disorders, including cerebral hemorrhage, traumatic brain injury, and subarachnoid hemorrhage, has demonstrated certain neuroprotective benefits [59, 60]. Research has indicated that phenothiazines can induce therapeutic hypothermia, mitigate cerebral edema, and thereby improve recovery rates in patients with severe traumatic brain injury. Previous clinical studies have established that a “cocktail” regimen comprising chlorpromazine (50 mg), promethazine (50 mg), and pentazocine (100 mg) significantly reduced mortality and improved neurological function scores in subarachnoid hemorrhage; here, pentazocine was primarily administered for pain management [49, 61]. Since patients with acute AIS typically do not require analgesia, the research focus has shifted to the neuroprotective effects of C + P independent of pentazocine. Earlier studies on severe cerebral hemorrhage also suggested that the neuroprotective effects of phenothiazines may operate partially independently of hypothermia induction, and it could substantially improve patient recovery rates [25, 49]. Historically, technological limitations have constrained C + P administration largely to intramuscular injection, slow intravenous push, or drip infusion, making precise control over drug onset and infusion rate challenging. The acute hypotension associated with rapid intravenous administration of these agents could lead to severe clinical complications and poor patient outcomes. Although studies have indicated that intravenous phenothiazines account for only a small proportion of significant hypotensive events [37, 58], the safety of this route remains debated. The advent of infusion pump technology has effectively addressed these concerns by enabling accurate control of drug concentration and infusion speed, thereby greatly enhancing the safety profile of intravenous phenothiazine administration in neurocritical care. A significant conceptual advance occurred in 2011 when five international professional associations formally proposed “targeted temperature management (TTM)” [62], underscoring the critical role of precise temperature control. Within this framework, the sedative effect of phenothiazines was initiated as part of the early phase of hypothermia therapy in a combined therapeutic strategy. One standardized protocol involves the administration of a mixture containing chlorpromazine (100 mg), promethazine (100 mg), pentazocine (150 mg), and saline to a total volume of 50 mL, delivered via a micro‐infusion pump at a rate of 0.5–4 mL/h. This method has been incorporated into hypothermia therapy guidelines and is now widely applied in clinical settings.

For AIS, the need to identify effective adjunctive neuroprotectants capable of shielding vulnerable neural cells represents a significant unmet clinical need. The ESCAPE‐NA1 trial, which utilized a neuroprotective peptide to salvage the ischemic penumbra with the goal of achieving neurological recovery at 90 days, exemplifies this endeavor. This study provides a novel therapeutic approach and has yielded promising results. However, the development and approval of new pharmacological agents face considerable challenges and require extensive clinical validation. In comparison, drug‐induced hypothermia coupled with metabolic suppression may represent a more economical, efficient, and straightforward strategic alternative [63, 64, 65].

In the broader field of AIS therapy, the exploration of drugs with hibernation‐like effects remains largely confined to the preclinical stage. Despite robust evidence of neuroprotection from animal studies, clinical research reports are exceedingly scarce. A phase I clinical trial conducted at Luhe Hospital in 2019 represents one of the few clinical investigations into C + P for AIS treatment. This study evaluated the oral administration of a low‐dose C + P regimen (25 mg + 25 mg, twice daily) in AIS patients, primarily focusing on safety and preliminary efficacy. While the treatment efficacy did not significantly differ between the oral C + P group and the control group, the trial established an initial safety profile for C + P administration in AIS patients [66].

Building upon those foundational safety data, the research team proposed a prospective study involving intravenous infusion of C + P (50 mg each, at a rate of 5 mL/h) in AIS patients. This subsequent investigation aimed to evaluate the safety and efficacy of C + P in patients receiving intravenous thrombolysis, thereby exploring the potential neuroprotective role in a clinical setting [8, 66]. Safety remains a paramount consideration. While the safety profile of phenothiazines for sedation in neurocritical care is well‐established, their application for inducing sub‐hypothermia and reducing metabolism in AIS patients requires specific vigilance. Particular attention must be given to their effects on vital signs, such as blood pressure and heart rate, especially during reperfusion therapy. Given the baseline cerebral hypoperfusion in AIS patients and the potential for synergistic effects when co‐administered with other anesthetic or sedative agents, a meticulously designed dosing regimen is imperative to avoid serious adverse consequences, including profound hypotension, aspiration, and exacerbation of cerebral ischemia [39, 67]. Furthermore, other drug‐specific side effects, such as allergic reactions and extrapyramidal symptoms, warrant careful monitoring.

## 5 Summary and Outlook

The exploration of neuroprotective agents as adjunctive therapies remains a major focus of contemporary stroke research [68]. The majority of clinical trials for neuroprotective strategies are designed to compare a drug against a placebo, often in patient cohorts that do not receive reperfusion therapy. It is critical to emphasize that reperfusion therapy remains the cornerstone of current treatment paradigms and may constitute a necessary condition for neuroprotective agents to demonstrate clinical efficacy. Consequently, investigating neuroprotectants in isolation from reperfusion may represent a suboptimal strategic direction [64].

Phenothiazines, particularly the C + P combination, present a more practical and cost‐effective solution, sustaining considerable interest as an adjunctive treatment. While their neuroprotective efficacy has been consistently demonstrated in preclinical models, a significant translational gap persists, especially in the context of AIS, where clinical data remain scarce. The ischemic penumbra—a region of viable but threatened brain tissue—constitutes a critical therapeutic target in stroke and holds immense potential for salvage. Future neuroprotective research will undoubtedly concentrate on this area. Existing preclinical evidence points to the potential of C + P to salvage the ischemic penumbra, reduce infarct volume, and improve neurological outcomes in animal models. However, substantial challenges remain in translating these effects into safe and effective clinical applications, particularly within the complex and time‐sensitive environment of emergency care. Overcoming these hurdles will require extensive and well‐designed clinical research.

In parallel, scientific interest extends to the concept of a hibernation‐like state. Although the concept itself is not new, standardized criteria for its definition are currently lacking. The core physiological manifestations of this state involve artificially induced hypothermia and hypometabolism; however, the underlying mechanisms are likely more complex, representing a condition that encompasses multiple integrated physiological features. Notably, a body of evidence suggests that C + P is capable of precisely inducing such a state. Nevertheless, further investigation is required to substantiate this effect and elucidate its full scope. In summary, C + P represents a candidate therapy of interest, with neuroprotective potential that warrants further investigation. However, rigorous clinical trials are essential to determine whether these preclinical findings can translate into meaningful benefits for patients with AIS. Future research efforts should be directed toward a deeper mechanistic elucidation and the advancement of rigorous clinical trials to validate and refine its therapeutic application.

## Author Contributions

All the authors have contributed significantly, and all the authors agree with the content of the manuscript and agree to its submission to CNS Neuroscience and Therapeutics.

## Funding

This work was supported by the National Natural Science Foundation of China (82027802, 82371470, 82071468, and 82201618).

## Conflicts of Interest

The authors declare no conflicts of interest.

## Data Availability

Data sharing not applicable to this article as no datasets were generated or analysed during the current study.
